# Young Coconut Juice Reduces Some Histopathological Changes Associated with Alzheimer's Disease through the Modulation of Estrogen Receptors in Orchidectomized Rat Brains

**DOI:** 10.1155/2019/7416419

**Published:** 2019-11-29

**Authors:** Tatcha Balit, Mosaad A. Abdel-Wahhab, Nisaudah Radenahmad

**Affiliations:** ^1^Department of Anatomy, Faculty of Science, Prince of Songkla University, Hat Yai, Songkhla, Thailand; ^2^Department of Food Toxicology and Contaminants, National Research Center, Dokki, Cairo, Egypt

## Abstract

*Propos*e. This study aimed to evaluate the protective role of young coconut juice (YCJ) against the pathological changes in Alzheimer's disease (AD) in orchidectomized (orx) rats. *Methods and Results*. Animals were divided into 7 groups including: baseline normal control group, sham control, orx rat group, orx rat group injected with 2.5 *μ*g/kg b.w. estradiol benzoate (EB) 3 days a week for 10 weeks, and the orx rat groups treated orally with 10, 20, and 40 ml/kg b.w. of YCJ for 10 weeks. At the end of treatment period, animals were sacrificed and the brain of each rat was removed, fixed in 10% neutral formalin, and stained by specific antibodies against NF200, parvalbumin (PV), *β*-amyloid (A*β*), and estrogen receptors (ER*α* and ER*β*). The results showed that the number of NF200- and PV-reactive neurons in the hippocampus and cerebral cortex was significantly reduced in orx rats. However, it restored to normal in orx rats injected with EB or those administrated with YCJ in a dose-related manner. Neurons containing *β*-amyloid (A*β*), a hallmark of Alzheimer's disease (AD), were found to be increased in the orx rats; however; they were reduced by EB injection or YCJ administration. These results suggested the binding of the YCJ active ingredient(s) with estrogen receptors (ERs) in the brain as indicated by the detection of ER*α* and ER*β* in neurons since a significant correlation was detected between NF200-/PV-reactive neurons vs ER*α*-/ER*β*-reactive neurons.*Conclusion*. It could be concluded that YCJ is effective as EB in reducing AD pathology, probably by being selective estrogen receptor modulators.

## 1. Introduction

With advancing age, a significant decrease in sex steroid hormones is occurred in men and women leading to the development of Alzheimer's disease (AD) [1, 2]. In males, the depletion of testosterone level affects different organs that are dependent on androgen, especially the brain. It was reported that the level of testosterone in the brain is inversely correlated with the brain levels of *β*-amyloid 1-42 (A*β*), which is well known as one of the AD hallmark pathologies [3]. This substance is accumulated as A*β* plaques consisting of small peptide fragments produced by abnormal proteolysis of a larger protein called amyloid precursor protein, which passes through the neuronal membrane and gets accumulated outside the neurons [4]. The A*β* plaques are increased in andropausal human brains [5, 6] and in the brains of aging male mice [7]. On the other hand, it was reported that female sex is considered as one of the major risk factors for the development of late-onset AD [8]. Epidemiologic studies have been documented that women comprise two-thirds of people living with AD, regardless of age and ethnicity [8–10]. The increased risk for AD in women as initiated by brain modifications during the menopause transition (MT) and the estrogen deficiency is well documented [11]. It was also found that estrogen supplement plays an important role in the protection of AD in male mice by significantly lowering A*β* levels in the Cornu ammonis 1 (CA1) area of the hippocampus [7].

One of the AD characteristics is the loss of either large-projection pyramidal neurons or small interneurons [12]. The molecular changes of the neurofilament-related proteins (NF200) in cytoskeleton of cells lead to AD pathologies [6]. Furthermore, parvalbumin (PV), a calcium-binding protein in GABAergic interneurons, also plays important roles in calcium homeostasis and thus prevents neuronal damage [13]. Both NF200 and PV are decreased in animal and human brains of neurodegenerative diseases [14, 15].

Although estrogen supplement could reduce AD pathologies in human brains, it could also cause gynecomastia and induce benign prostatic hyperplasia and prostatic cancer [16]. Therefore, plant-derived phytoestrogens maybe better choice than estrogens. Similar to 17*β* estradiol (E2), phytoestrogens can also bind to *α* and *β* estrogen receptors (ER*α* and ER*β*) [17]. These two types of estrogen receptors present in both human and rat brains [18] and ER*β* were predominant in the cerebral cortex and the hippocampus, the areas that are playing an important role in learning and memory [19]. Both E2 and phytoestrogens could pass through neurilemma to bind with cytoplasmic estrogen receptors [17]. This binding prevents glucose oxidase-induced oxidative stress and lipid peroxidation that lead to degenerative changes in neurons [17, 20, 21].

In our previous works, we reported that giving YCJ to ovariectomized rats could increase NF200- and PV-reactive cells in the brain and reduce the accumulation of A*β* [15, 22]. The active ingredient(s) of YCJ was found to be *β*-sitosterol [23]. YCJ was found to prevent osteoporosis [24, 25], preserve cells involved in motility of the gastrointestinal tract [26], accelerate wound healing, and improve skin complexion [27]. It was also found that YCJ at a dose of 100 ml/kg b.w./day could reduce AD pathologies in ovariectomized rats within 4 weeks. However, high dose of YCJ supplement had caused unfavorable side effects, such as glycogen deposition in the liver. Therefore, the aim of the current study was to evaluate the protective role of YCJ against pathological changes in the brain of orx male rats in addition to its effect on the ovariectomized rats using the immunohistochemistry staining of NF200 and PV reactivities as they represent pyramidal and nonpyramidal neurons of the brain, respectively, as well as A*β*-reactive neurons as an index of A*β* accumulation in the brain. Moreover, the number of ER*α*- and ER*β*-reactive neurons was also determined to reveal if YCJ could act through the stimulation of these receptors.

## 2. Materials and Methods

### 2.1. YCJ Preparation

Young coconuts (*Cocos nucifera* L., *Arecaceae*) were collected from Khlong Hoi Khong District, Hat Yai, Songkhla, Thailand. The preparation of YCJ was carried out as described in our previous work [28]. Fresh YCJ was freeze-dried (100 ml produces 6 g powder) and the powder was kept at −30°C and freshly reconstituted before oral feeding of the rats.

### 2.2. Animals

Adult male Wistar rats (8 months old and 250–300 g b.w.) were purchased from Mahidol University, Salaya campus. The animals were maintained on standard pellet food housed in a room free from any source of chemical contamination, artificially illuminated (12 h dark/light cycle), and thermally controlled (25 ± 1°C) and humidity (50 ± 5%) at the Animal House Laboratory, Faculty of Science, Prince of Songkla University, Hat Yai, Songkhla, Thailand. All animals received humane care in compliance with the guidelines of the Animal Care and Use Committee of Prince of Songkla University and the National Institutes of Health (NIH publication 86-23 revised 1985. The protocol was approved under the license number 01/59).

#### 2.2.1. Procedure for Orchidectomy

Each rat was anesthetized and laid supine on the operating table. Scrotal shaving was carefully performed and 70% alcohol followed by Betadine was applied for a septic prevention. A median incision about 1.0 cm long was made through the skin at the median line between 2 scrotums. The cremaster muscles were cut with a small incision of about 7 mm. Using the blunt forceps, the testicular fat pad was pulled out through the incision. The epididymis was pulled out along with the testis, followed by the vas deferens and the blood vessels of the testicles. The vas deferens and blood vessels were ligated and cut and therefore, the testis could be removed safely. Muscle and skin were carefully sutured layer by layer, and Betadine was again applied on the wound as aseptic prevention. The rats were kept warm under the reading lamp in a controlled room temperature 25 ± 2°C until they recovered and returned to the animal house keeping.

#### 2.2.2. Experimental Design

After an acclimatization period of 1 week, the animals were divided into seven groups (10 rats/group) and treated for 10 weeks as follows: group 1, baseline normal control (NC) animals, were sacrificed on the first day of experiment without any treatment; group 2, sham-operated rats (SC), received reverse-osmosis water; group 3, orchidectomized rats (orx), received reverse-osmosis water; group 4, orx rats, intraperitoneally injected 3 days a week with 2.5 *μ*g/kg b.w. estradiol benzoate [11, 18]; and groups 5, 6, and 7, orx rats orally treated with YCJ at low (LD; 10 ml/kg b.w.), medium (MD; 20 ml/kg b.w.), and high dose (HD; 40 ml/kg b.w.), respectively. At the end of the treatment period, all animals were sacrificed by cervical dislocation and the whole brain of each animal was removed, fixed with 10% neutral formalin, paraffin processing, sectioning, and immunohistochemical staining.

### 2.3. Immunohistochemical Staining

Fourteen 5 *μ*m-thick sections from each block were prepared for cresyl violet staining and immunostaining as shown in Table 1. For immunostaining, the glass slides were coated by poly-L-lysine solution. The first two sections were stained with cresyl violet, and were used for light microscopy examination and anatomical orientation. The immunostaining technique was as follows. Sections were left on a hot plate at 37°C overnight followed by passing the sections through serial steps of fresh xylene and alcohol gradients. The sections were then left in distilled water for 10 min before antigen retrieval by microwave for half an hour was performed (0.01% sodium citrate antigen retrieval solution; 650 W). The sections were then allowed to cool down at room temperature for 15 min, followed by washing with Tris-buffered saline and incubation for 10 min with 0.3% H_2_O_2_ in methanol to quench any endogenous peroxidase activity. In the case of staining for A*β* 1–42, the sections were also pretreated for 30 min with 80% formic acid at room temperature. Having accomplished the preparatory phase, we then incubated the sections with the primary antibodies for 30 mins in a humidified chamber, washed them with Tris-buffered saline, and then incubated them with the secondary and tertiary antibodies with frequent Tris-buffered saline washes in between. The procedure was followed according to the steps provided with the ABC kit used (Vectastain® Elite ABC kit; Vector Laboratories, Burlingame, CA, USA). The substrate chromogen (1 min incubation time) used in the present study was diaminobenzidine enhanced by the addition of a nickel solution from the staining kit (Vector Laboratories). Sections were then counterstained with Mayer's haematoxylin for 5 seconds and cover slipped [15, 22]. Sections of uterus and ovary from normal female rats were used as positive controls for ER*α* and ER*β* immunostaining, respectively.

#### 2.3.1. Quantitative Analysis of Immunoreactive Cells

The total number of immunoreactive cells from the cerebral cortex (prefrontal, parietal, temporal, and occipital areas) and the hippocampus were counted under light microscopy (LM) with 40x magnification power. The counting was performed by two independent observers on 10 random fields of each slide using an image analysis system (Samba microscopic image processor; Samba Technologies, Meylan, France). Readings from 3 sections pertaining to each antibody were averaged and expressed as the mean number of immunoreactive cells/mm^2^.

### 2.4. Statistical Analysis

Shapiro–Wilk test was applied to test the normal distribution. Statistical analysis was performed using the one-way ANOVA followed by the LSD test available in the statistical program SPSS version 16.0 (SPSS, Inc., Chicago, IL, USA). Altman's nomogram was used for calculations of sample size. Random selection of the microscopic fields was achieved using a computer-generated list of random numbers (Excel version 5.0). Results were expressed as mean ± SEM, and *p* < 0.05 was considered significant.

## 3. Results

**Figure 1:**
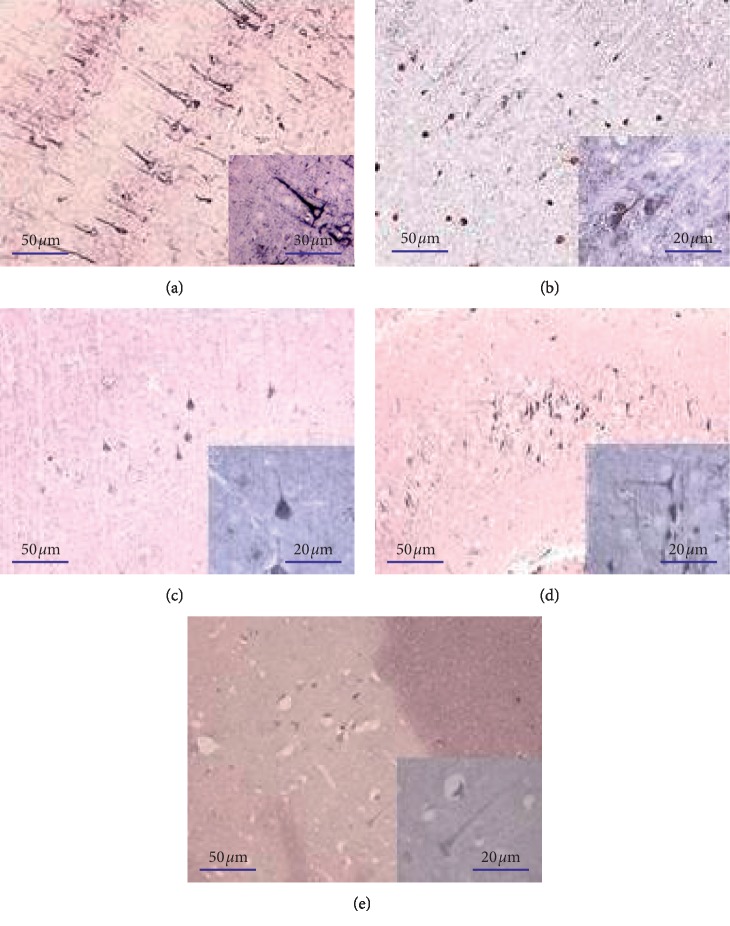
Examples of reactive neurons of YCJ (HD)-treated group: (a) NF200-reactive neurons in the parietal lobe, (b) PV-reactive neurons in the parietal lobe, (c) ER*α* reactive neurons in the prefrontal area, (d) ER*β*-reactive neurons in the hippocampus of the brains, and (e) A*β*-reactive neurons in the temporal lobe of the brain of the orx group.

The results presented in Figure 1 showed some examples of neurons positive for neurofilament 200 (NF200), parvalbumin (PV), estrogen receptor-*α* (ER*α*), and estrogen receptor-*β* (ER*β*) in the brain specimens of orx rats that received YCJ (HD) and the neurons positive for amyloid *β* (A*β*) in the brain specimen of the orx group. It is clear that the NF200-reactive neurons (Figure 1(a)) were predominantly localized in layers III and V of the cerebral cortex and in CA3, but not in CA1 and CA2 of the hippocampus. Positive reaction was observed in the triangular cell bodies and prominently in the dendrites and axon. The PV-reactive neurons were found in CA1, CA2, and CA3 of hippocampus and in all tested areas of the cerebral cortex. The positive reaction was observed prominently in the cell bodies and axons, but lightly in the dendrites (Figure 1(b)). Both ER*α*-reactive (Figure 1(c)) and ER*β*-reactive (Figure 1(d)) neurons were observed, predominantly in layers III and V of the cerebral cortex, and the reaction was in the cell bodies, dendrites, and axons. The A*β*-reactive neurons (Figure 1(e)) were detected in layers III and V of the cerebral cortex with more intensity in layer IV and also in CA3. The reaction was observed in the cytoplasm and partially present in the basal dendrites of pyramidal cells, but not in the nucleus. Besides neurons, A*β* signal was also observed in the choroid plexus.

**Figure 2:**
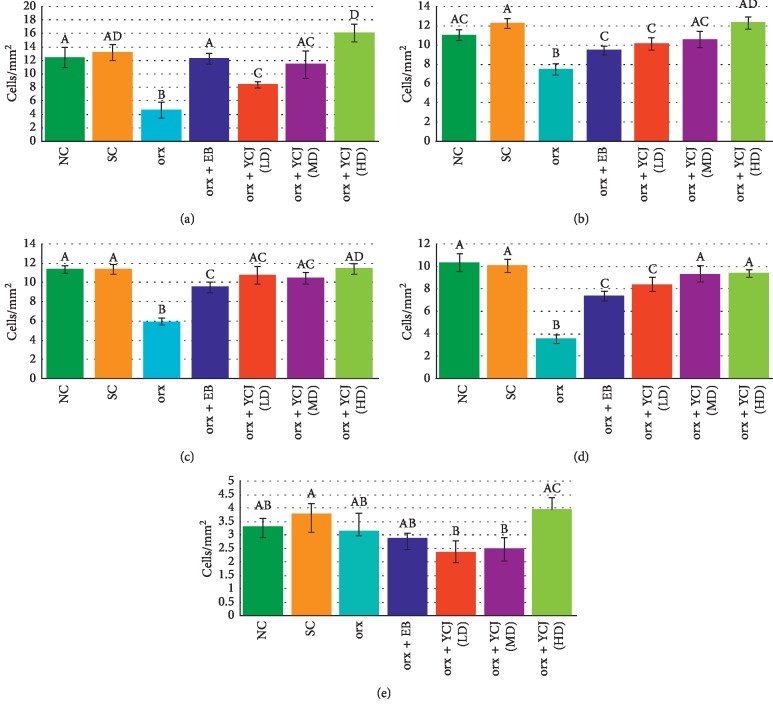
The numbers of NF200-reactive neurons in the Cornu ammonis (CA) of the (a) CA3 area, (b) prefrontal area, (c) parietal area, (d) temporal area, and (e) occipital area of the hippocampus of different groups. Columns superscript with different letters are significantly different at *p* < 0.05.

The data presented in Figure 2 show the number of NF200-reactive neurons in various areas of the brain under study. In the normal control (NC) and sham control (SC) groups, the number of positive NF200-reactive neurons was observed at high frequency in CA3 (Figure 2(a)), prefrontal (Figure 2(b)), parietal (Figure 2(c)), and temporal areas (Figure 2(d)), but much lower in the occipital area (Figure 2(e)). It is clear that in orx rats, the numbers of NF200-reactive neurons were significantly dropped in all brain areas tested, except in the occipital area. However, the recorded values were increased in orx rats treated with EB, and the number was restored to the normal level only in the CA3 and occipital cortex. Orx rats that received YCJ at the three tested doses showed a significant increase in the number of the reactive cells in a dose-related manner, and the orx rats that received the high dose were comparable to those of the control groups (NC and SC groups).

**Figure 3:**
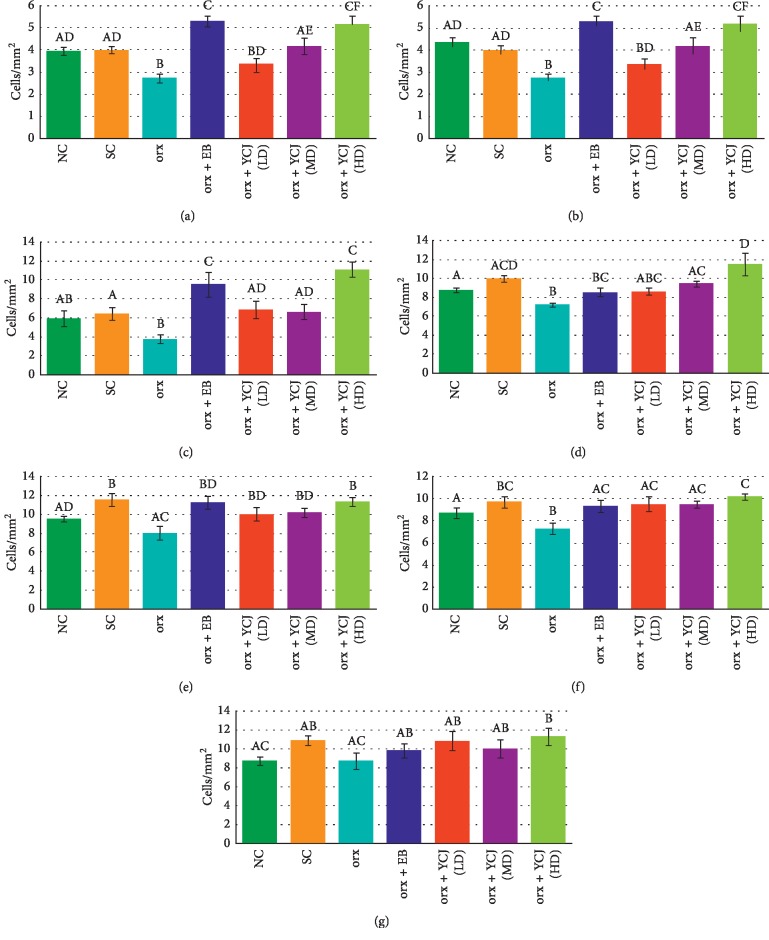
The numbers of PV-reactive neurons in the Cornu ammonis (CA) of the (a) CA1 area, (b) CA2 area, (c) CA3 area, (d) prefrontal area, (e) parietal area, (f) temporal area, and (g) occipital area of the hippocampus of different groups. Columns superscript with different letters are significantly different at *p* < 0.05.

The effect of different treatments on the number of PV-reactive neurons in different treatment groups is presented in Figure 3. PV-reactive neurons were found more in the cerebral cortex, compared to those in the hippocampus. In the hippocampus, CA3 seemed to contain more PV-reactive neurons than in CA1 and CA2. The number of PV-reactive neurons significantly decreased in CA1 (Figure 3(a)), CA2 (Figure 3(b)), CA3 (Figure 3(c)), prefrontal area (Figure 3(d)), parietal area (Figure 3(e)), temporal area (Figure 3(f)), and occipital area (Figure 3(g)) of orx rats. Treatment with EB or YCJ increased the number of PV-reactive neurons in all brain areas and even overshoot above normal in some brain areas, e.g., in the hippocampus (CA1, CA2, and CA3) and in the orx rats treated with YCJ (HD), which were comparable to those treated with EB.

**Figure 4:**
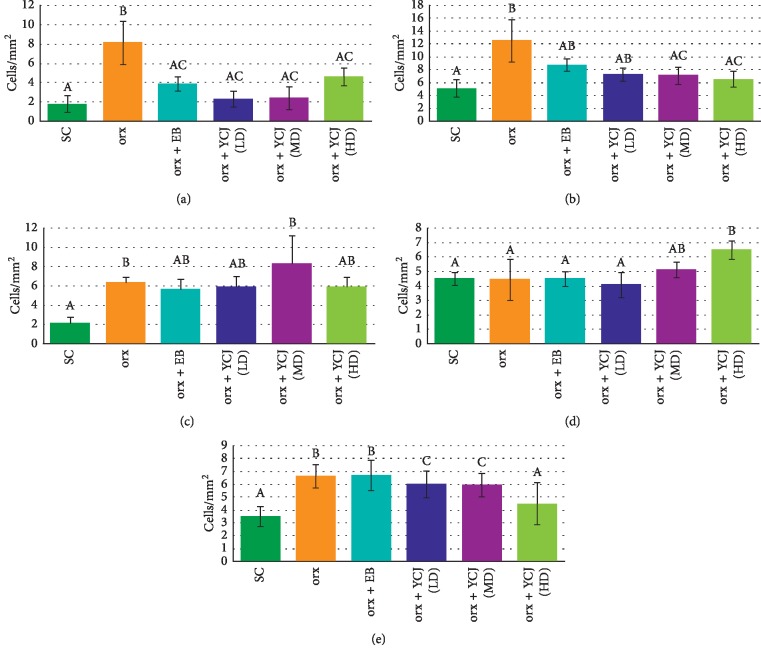
The numbers of A*β*-reactive neurons in the (a) Cornu ammonis, CA3, (b) prefrontal area, (c) parietal area, (d) temporal area, and (e) occipital area of the hippocampus and areas of cerebral cortices in all different treatment groups. Columns superscript with different letters are significantly different at *p* < 0.05.

Regarding the number of A*β*-reactive neurons in various brain regions (Figure 4), unfortunately, the A*β*-reactive neurons in the brain of normal control (NC) group were missed being due to technical errors during paraffin sectioning process. Therefore, the obtained data were compared with the sham control (SC) group. In orx rats, the number of A*β*-reactive neurons was significantly increased in CA3 (Figure 4(a)), prefrontal area (Figure 4(b)), parietal area (Figure 4(c)), temporal area (Figure 4(d)), and occipital area (Figure 4(e)). Treatment with EB or YCJ at the three tested doses decreased the number of A*β*-reactive neurons in orx rats, although the number was still high in all groups compared to SC rats. The levels in any groups of YCJ treatments did not differ statistically, suggesting that even the lowest dose of YCJ (LD) was adequate to bring back the number of A*β*-reactive neurons to normal following orchidectomy. Moreover, the high dose of YCJ was the most effective (except in the temporal area) and showed the best improvement in the number of A*β*-reactive neurons.

**Figure 5:**
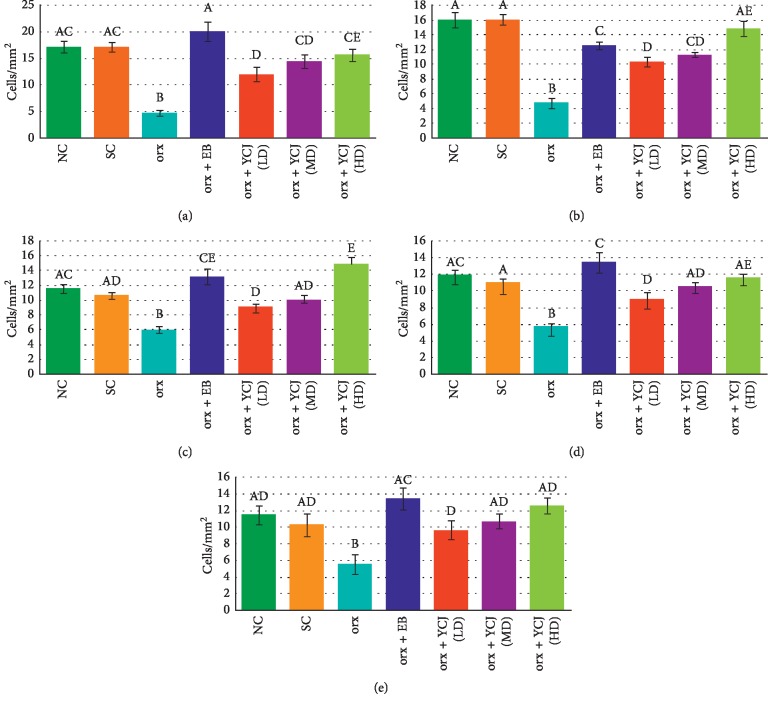
The numbers of ER*α*-reactive neurons in the (a) Cornu ammonis, CA3, (b) prefrontal area, (c) parietal area, (d) temporal area, and (e) occipital area of the hippocampus and areas of cerebral cortices in all different treatment groups. Columns superscript with different letters are significantly different at *p* < 0.05.

**Figure 6:**
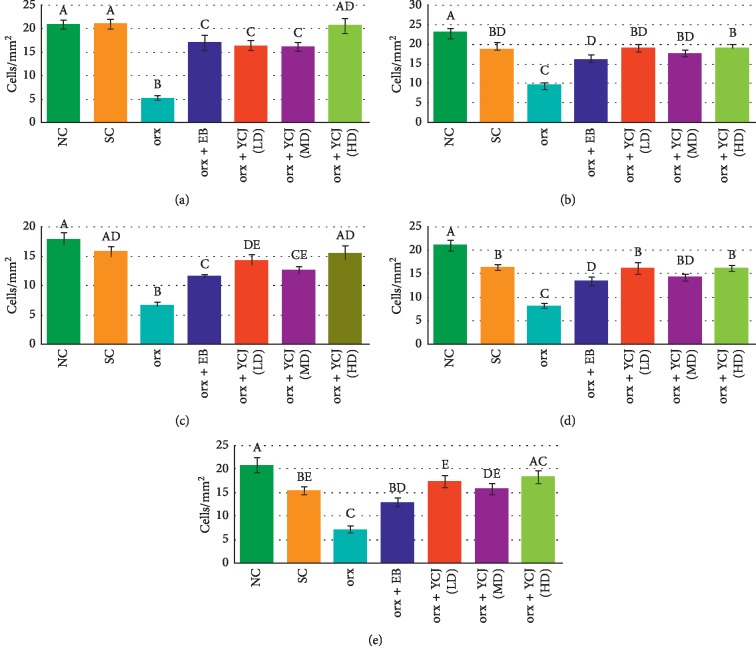
The numbers of ER*β*-reactive neurons in the (a) Cornu ammonis, CA3, (b) prefrontal area, (c) parietal area, (d) temporal area, and (e) occipital area of the hippocampus and areas of cerebral cortices in all different treatment groups. Columns superscript with different letters are significantly different at *p* < 0.05.

Following orchidectomy, the numbers of ER*α*-reactive neurons (Figure 5) were decreased significantly in CA3 (Figure 5(a)), prefrontal area (Figure 5(b)), parietal area (Figure 5(c)), temporal area (Figure 5(d)), and occipital area (Figure 5(e)). The number of ER*α*-reactive neurons in CA3 of the brain of the orx animals treated with EB was comparable to the control in all treated areas except the prefrontal area, which was still lower than the control. The orx rats treated with YCJ at the three tested doses showed a significant improvement in the number of ER*α*-reactive neurons and all areas were similar to the control. In general, the number of ER*β*-reactive neurons was higher than that of ER*α*-reactive neurons in all tested areas. The numbers of ER*β*-reactive neurons in all brain areas examined (Figure 6) were significantly decreased after orchidectomy (Figures 6(a)–6(e)). Sham operation of orchidectomy (SC group), however, had effect on the numbers in the prefrontal, the temporal, and the occipital areas by reducing the number of the reactive neurons significantly compared to that of the NC group. EB administration to orx rats improved the number ER*β*-reactive neurons in all tested areas, although this treatment did not normalize the number of ER*β*-reactive neurons. Supplement with YCJ to orx rats induced a significant improvement in the number of ER*β*-reactive neurons in a dose-dependent manner, and YCJ (HD) was the most effective dose.

**Figure 7:**
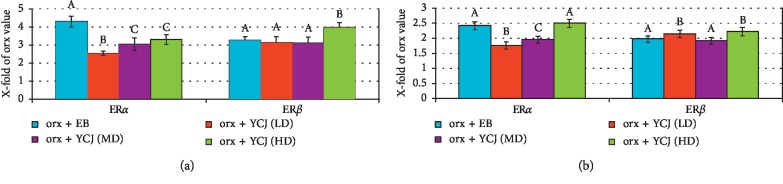
The increase of the numbers of ER*α*- and ER*β*-reactive neurons in the CA3 area (a) and cerebral cortex (b) of the rats after EB and YCJ treatments, compared to the numbers of the respective neurons in the orx rats (oc group). Values in the cerebral cortex are averages of those in the prefrontal, parietal, temporal, and occipital areas combined. In ER*α* and ER*β*, columns superscript with different letters are significantly different at *p* < 0.05.

The effects of EB and YCJ treatments on the numbers of ER*α*- and ER*β*-reactive neurons in the hippocampus (CA3, Figure 7(a)) and all areas of the cerebral cortex (Figure 7(b)) compared to the mean numbers of their respective neurons in the orx group are presented in Figure 7. ER*α*-reactive neurons in the CA3 area was increased more than fourfolds compared with the orx group and 2.5–3.3 times in the groups treated with YCJ at the three tested doses. This result suggests that YCJ might not be as efficient as EB in restoring the number of ER*α*-reactive neurons in the CA3 area. On the contrary, the number of ER*β*-reactive neurons was increased in the CA3 area of orx rats treated with YCJ compared to EB. This result suggested that YCJ may be effective than EB in restoring ER*β*-reactive neurons in orx rats. The current results also indicated that the numbers of ER*α*- and ER*β*-reactive neurons in orx treated with EB and YCJ were comparable in all areas tested (i.e., the frontal, parietal, temporal, and occipital areas). This increase reached 2.0- to 2.5-fold compared to that of the orx plus EB group. Moreover, no significant difference was observed between the numbers of ER*α*- and ER*β*-reactive neurons.

**Figure 8:**
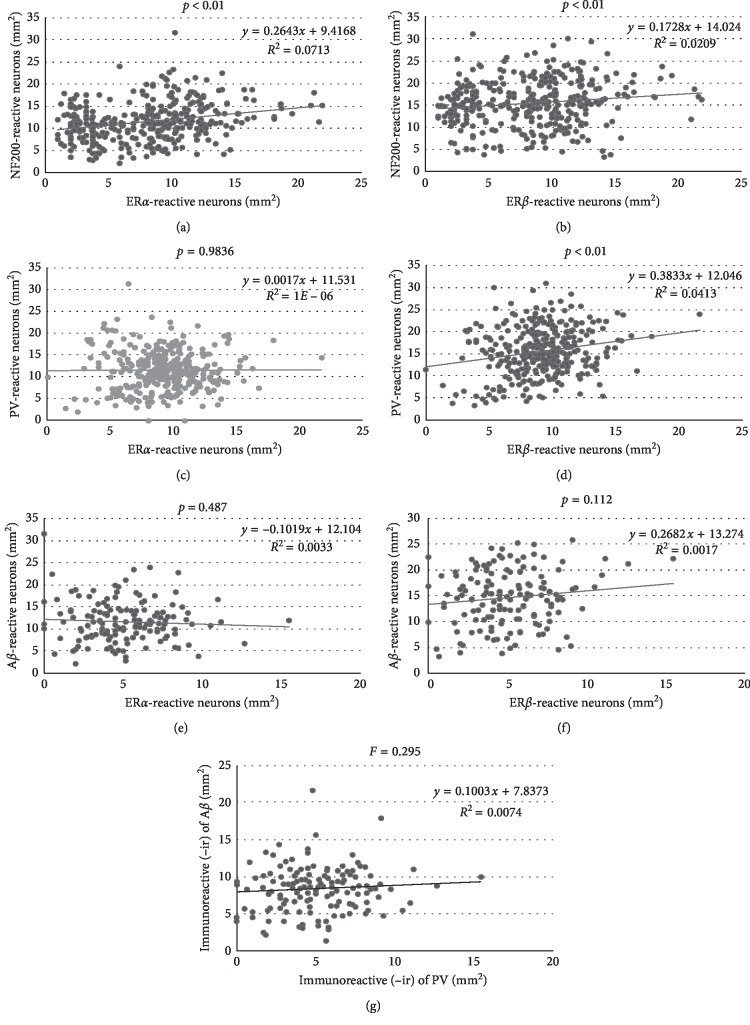
Plot of numbers of the NF200-reactive neurons against the numbers of ER*α*-reactive neurons (a) and ER*β*-reactive neurons (b), the PV-reactive neurons against the numbers of ER*α*-reactive neurons (c) and ER*β*-reactive neurons (d) and the A*β*-reactive neurons against the numbers of ER*α*-reactive neurons (e) and ER*β*-reactive neurons (f), and (g) correlation between PV neuron density with A*β*-reactive neurons from the same rats and from all animal groups.

The correlation between the numbers of NF200-, PV-, and A*β*-reactive neurons and the numbers of ER*α*- and ER*β*-reactive neurons was evaluated in the same animal regardless of groups (Figure 8). The results revealed that the numbers of NF200-reactive neurons were positively correlated with either ER*α*- or ER*β*-reactive neurons, while those of PV-reactive neurons were positively correlated with ER*β*-reactive neurons, but not ER*α*-reactive neurons. On the contrary, the numbers of A*β*-reactive neurons were not correlated with ER*α*- or ER*β*-reactive neurons. Additionally, no correlation was found between PV-ir cell numbers and A*β*-reactive neurons at *F* = 0.295 (Figure 8(g)).

**Figure 9:**
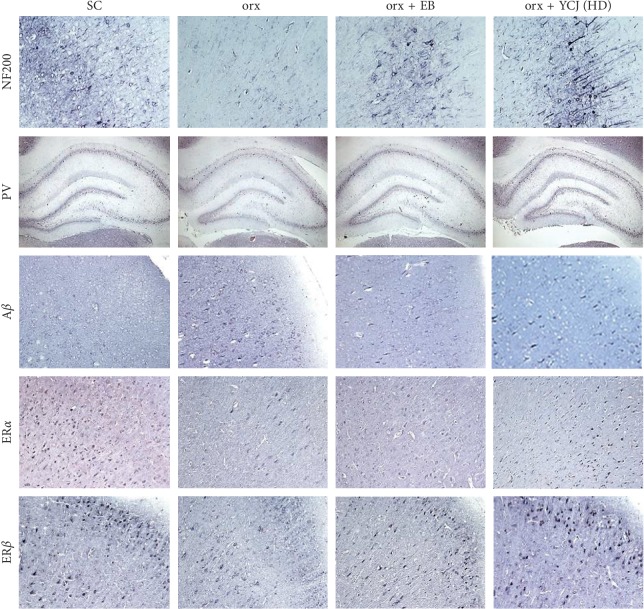
Photomicrograph showing immunohistochemistry of NF200-, A*β*-, ER*α*-, and ER*β*-reactive neurons from the parietal area of the cerebral cortex and PV-reactive neurons from the hippocampus of the SC group, orx control, orx rats + EB, and orx rats + YCJ (HD). The same scale is used for all pictures except for PV-reactive neurons.

The results of immunohistochemistry of brain areas in sham control, orx, orx plus EB, and orx plus the high dose of YCJ are depicted in Figure 9. NF200-, A*β*-, ER*α*-, and ER*β*-reactive neurons were found in the parietal area of the cerebral cortex; however, PV-reactive neurons were found in the hippocampus. It was visually evident that NF200- and ER*β*-reactive neurons were depleted in orx rats and were restored to normal features in orx plus EB group and orx plus the high dose of YCJ group. The restorative feature of the ER*α*-reactive neurons in orx rats was less obvious than those of NF200- and ER*β*-reactive neurons, although it was visible. The features of PV-reactive neurons in orx rats and those treated with EB or YCJ were not clearly observed. However, the features of the increase in A*β*-reactive neurons in orx rats were visually decreased by EB and YCJ treatments.

## 4. Discussion

AD is the sixth causative factor of death worldwide with a long prodromal and preclinical phase reach about 20 years and an average of 8–10 years clinical duration. Currently, no treatments are available to stop, slow, or reverse the progression of the disease process [29, 30]. The results of the current study revealed that orx rats showed severe disturbances in all types of the reactive neurons in the brain including NF200, PV, A*β*, ER*α*, and ER*β*. The number of NF200-reactive neurons in orx rats was decreased in all tested areas of the brain except the occipital area. Similar results were reported previously in rats during testosterone deficiency suggesting the important role of testosterone in preventing AD [31]. Furthermore, disorders interrupting the secretion of testosterone may trigger apoptosis in neural tissue in either rat [32] or human brains [33]. The presence of testosterone was reported to prevent A*β* accumulation in neurons by binding to the androgen receptors in mice [7] and rats [34]. Although the effect of testosterone may not involve the androgen receptor directly, it was found to increase luteinizing hormone level in the brain, which resulted in a decrease in testosterone feed-back loop and consequently enhanced A*β* accumulation [7]. The same authors reported that the A*β* accumulation in the subiculum and amygdala of the hippocampus in orx mice could be reversed by EB treatment, suggesting that estrogen could prevent AD pathologies in male mice as well [7]. Moreover, Ramsden et al. [34] reported that age-related androgen depletion may result in accumulation of A*β* in the male brain and thereby act as a risk factor for the development of AD. These results supported the results reported in the current study and confirmed that EB treatment lowered the number of A*β*-reactive neurons in the CA3 and the prefrontal area.

In a previous work, we reported that YCJ contains 58% of its composition *β*-sitosterol besides other sterols including stigmasterol, stigmastatrienol, *α* spinasterol, and fucosterol [23]. Moghadasian [35] reported that *β*-sitosterol has a structure similar to the animal cholesterol and so, it may act as a sex steroid precursor. Moreover, the methanol extract of coconuts showed an estrogenic effect in rats due to its high content of *β*-sitosterol, stigmasterol, and other flavonoids [36]. Previous reports indicated that coconut extract is rich in phytohormones such as abscisic acid (ABA), auxin, gibberellins (GAs), and other cytokinins [37–39]. One of the most important cytokines in coconuts is *trans*-zeatin, which showed a potential inhibitory action on acetylcholinesterase and was reported to be effective for the treatment of AD and its related dysfunctions [40, 41]. Furthermore, trans-zeatin prevents the formation of amyloid *β*-protein, which has an important role in the development and progress of AD [42] and exhibited antiaging effects on fibroblast cells of humans [43]. In the current study, the protective role of YCJ against the disturbances of different reactive neurons in the brain of orx rats is mainly due to its strong estrogenic effect, which facilitates the synthesis of endogenous estrogens.

The positive correlation between NF200-reactive cells and ER*α*- or ER*β*-reactive cells reported in the current study suggested that some of the active ingredients of YCJ may bind with either ER*α* or ER*β*. However, the positive correlation between the number of PV-reactive cells and ER*β*-reactive cells suggests that these active ingredients may bind ER*β*, but not ER*α*. Additionally, several reports indicated that treatment with estrogen increased PV-immunoreactivity in the rat frontal cortex and contributed to the formation the dendritic spine of the PV-reactive neurons [44, 45]. Moreover, estrogen also affected the dopaminergic system by intrinsically mediated mechanism through its antioxidant properties, such as by regulating Ca^2+^ homeostasis in the dopaminergic neurons and subsequently enhancing PV expression [44] through ER-dependent or ER-independent mechanisms [46]. On the other hand, estrogen deficiency is associated with the increase in proinflammatory cytokines, such as interleukin (IL)-1, IL-6, and tumor necrosis factor-*α*, which is linked to the development of AD or neurodegenerative disease [2]. In the same concern, Yue et al. [47] demonstrated a robust reduction of brain 17*β*-estradiol levels in frontal cortex homogenates prepared from rapidly acquired postmortem brain tissue of women with AD. Moreover, the clinical trial of estrogen-plus-progestin therapy failed to demonstrate any beneficial effect on treating AD symptoms [48]; much evidence indicates that estrogen plays neuroprotective roles in the brain [49, 50].

The current results also revealed that the number of A*β*-reactive cells was not correlated with the number of ER*α*- and ER*β*-reactive cells. These results are in agreement with the previous reports, which suggested that the level of *β*-amyloid was upregulated by luteinizing hormone and it was increased by a lack of estrogen feedback at the hypothalamic or pituitary levels [7, 26, 51]. Several reports indicated that phytoestrogens may act as a selective estrogen receptor modulator (SERM) in the brain and other tissues, such as keratinocytes, fibroblasts, and sebaceous gland [26, 27, 52]. They have been shown to have higher affinity to ER*β* than to ER*α* [27, 53]. Consequently, these substances may bind to ER*α* and ER*β* and act as either agonists or antagonists depending on the pharmacological compounds present as well as on the target tissues [54]. Two hallmarks of AD are amyloidosis and hyperphosphorylated tau. Since the present work is a preliminary study, the hyperphosphorylated TAU protein deposits in the brain of orchidectomized rat brains treated with young coconut juice will be considered in our future work.

## 5. Conclusion

The results of this study revealed that: (1) orx rats showed pathologies due to the deficiency of testosterone hormone, (2) YCJ treatment, at various doses tested, restored the decreased numbers of NF200-, PV-, ER*α*-, and ER*β*-reactive neurons caused by orchidectomy to normal or close-to-normal levels, (3) the increasing number of A*β*-reactive neurons following orchidectomy was also suppressed by YCJ treatment, and (4) the effects of YCJ were comparable to those of EB treatment. In most cases, the dose of YCJ at 40 ml/kg b.w. was the best. These findings suggest that the potential therapeutic property of YCJ in preventing AD pathology in male rats is caused by a lack of testosterone.

## Figures and Tables

**Table 1:** Antibodies, concentration, and manufacturer used for brain sections of each rat.

Section No.	Staining
1-2	Cresyl violet, for histological orientation
3-4	Anti-NF200 antibody, 1 : 2,000 diluted (N0142, Sigma-Aldrich, USA)
5-6	Anti-PV antibody, 1 : 1,000 diluted (P3088, Sigma-Aldrich, USA)
7-8	Anti-*β*-amyloid 1-42 polyclonal antibody, 1 : 50 diluted (AB5078P, Chemicon, USA)
9-10	Mouse anti-estrogen receptor *α* (aa-120-170) antibody, 1 : 500 diluted (MAB447, Chemicon international, USA)
11-12	Estrogen receptor *β* antibody, 1 : 2,000 diluted (PA1-310B, Thermo Fisher Scientific, USA)
13-14	Immunostaining, omitting primary antibodies (negative control)

## Data Availability

The data used to support the findings of this study are available from the corresponding author (Dr. Nisaudah Radenahmad) upon request.

## Abbreviations

AD: Alzheimer's disease
orx: Orchidectomized
EB: Estradiol benzoate
NF200: Neurofilament 200
PV: Parvalbumin
ER*α*: Estrogen receptor-*α*
ER*β*: Estrogen receptor-*β*
A*β*: Amyloid *β*-protein
SERMs: Selective estrogen receptor modulators
CA1: Cornu ammonis 1
YCJ: Young coconut juice.
